# “First do no harm”: a clinical case of severe and enduring anorexia nervosa

**DOI:** 10.1186/2050-2974-3-S1-O34

**Published:** 2015-11-23

**Authors:** Emma Thomas, Daniela Alloro

**Affiliations:** 1 Tauranga Hospital, New Zealand

## 

Anorexia Nervosa is a devastating disorder with the highest mortality in mental health. For some, it becomes a life long struggle with immense personal and social consequences and premature death. Severe and enduring anorexia is often synonymous with intractable disorder.

This presentation challenges the common definition of treatment resistant disorder. It presents the case of a 60 year old woman who has lived with the disorder for over 30 years. The treatment went beyond weight restoration and resolution of the anorexic thinking; the main focus was on minimising harm and focusing on best possible quality of life as defined in a collaborative therapeutic relationship with the client. The presentation is not only about what went well but also trial and error and learning to be flexible and inventive rather than being stuck with pre-conceived ideas of ‘successful’ treatment goals.

